# Gefitinib (ZD1839, Iressa™) as palliative treatment in recurrent or metastatic head and neck cancer

**DOI:** 10.1038/sj.bjc.6602999

**Published:** 2006-02-21

**Authors:** A M Kirby, R P A'hern, C D'ambrosio, M Tanay, K N Syrigos, S J Rogers, C Box, S A Eccles, C M Nutting, K J Harrington

**Affiliations:** 1 Head and Neck Unit, Royal Marsden Hospital, 203 Fulham Road, London, UK; 2 Department of Statistics, Royal Marsden Hospital, 203 Fulham Road, London, UK; 3 McElwain Laboratories, Institute for Cancer Research, 15 Cotswold Road, Sutton, UK; 4 Targeted Therapy Laboratory, Institute of Cancer Research, Cancer Research UK Centre for Cell and Molecular Biology, Chester Beatty Laboratories, 237 Fulham Road, London SW3 6JB, UK

**Keywords:** gefitinib, head and neck cancer', palliation

## Abstract

To assess the level of activity and toxicity of gefitinib (ZD1839, Iressa™) in a population of patients with locally recurrent and/or metastatic head and neck cancer. Patients were recruited into an expanded access programme through the multidisciplinary head and neck clinics at the Royal Marsden and St George's Hospitals. Patients were required to have received at least one course of standard systemic chemotherapy or radiation therapy, or be medically unfit for chemotherapy. Patients were commenced on single-agent gefitinib at a dose of 500 mg day^−1^. Clinical, symptomatic and radiological response, time to progression (TTP), survival and toxicity were recorded. A total of 47 patients were enrolled (35 male and 12 female) with a median age of 62 years (range 18–93 years). The observed clinical response rate was 8% with a disease control rate (complete response, partial response, stable disease) of 36%. In all, 34% of patients experienced an improvement in their symptoms. The median TTP and survival were 2.6 and 4.3 months, respectively. Acneiform folliculitis was the most frequent toxicity observed (76%) but the majority of cases were grade 1 or 2. Only four patients experienced grade 3 toxicity of any type (all cases of folliculitis). Gefitinib was well tolerated and yielded symptomatic improvement in one-third of patients. However, this agent appeared to possess limited antitumour activity in this group of patients with head and neck cancer in whom the objective response rate, median TTP and survival were all lower than has been reported in a previous study.

Head and neck cancer is the sixth most common malignant tumour diagnosed in Europe. More than 100 000 cases were diagnosed in Europe in 2000 with more than 50 000 deaths occurring in the same year. Many patients present with locally advanced, unresectable (stages III and IV) disease in which the standard treatment is a combination of platinum-based chemotherapy and radiotherapy. For patients who relapse after such treatment, the only curative treatment option is surgical. If this is not possible, as is often the case, platinum-based chemotherapy is frequently used as palliative therapy (Forastiere *et al*, 1992; Jacobs *et al*, 1992) Other drugs that have some activity in this tumour type include bleomycin, methotrexate, taxanes, gemcitabine and vinorelbine (Clark *et al*, 2001; Basaran *et al*, 2002). However, tumour response rates rarely exceed 30–35% (Liggett and Forastiere, 1995) and responses are usually of short duration such that the outlook in the setting of recurrent/metastatic disease is poor, with 1-year survival below 30% and median survival only 4 months. Treatment options for patients with progressive disease (PD) are limited and, therefore, this is an area of high unmet need.

The epidermal growth factor receptor (EGFR), a member of the erbB family of receptors, is a transmembrane glycoprotein whose intracellular domain has tyrosine kinase activity. Activation of the EGFR increases the proliferation, differentiation and survival of cancer cells via multiple phosphorylation-dependent signalling cascades down to transcription factors in the nucleus. Epidermal growth factor receptor is expressed at low levels on the surface of normal cells. However, it is implicated in the development of various malignancies and is overexpressed in 30% of human solid tumours and up to 90% of squamous cell cancers of the head and neck (Dassonville *et al*, 1993). Epidermal growth factor receptor is also implicated in angiogenesis, ability to metastasise, and inhibition of apoptosis (Baselga, 2000; Shintani *et al*, 2003a, 2003b) Indeed, EGFR expression has been found to relate closely to prognosis in head and neck cancer, higher levels correlating with poorer progression-free and overall survival (Nicholson *et al*, 2001).

Gefitinib (ZD1839, Iressa™) is an orally active, selective EGFR-tyrosine kinase inhibitor that blocks the signal transduction pathways described above. In human head and neck cancer cell lines it has been shown to inhibit cell proliferation in a dose-dependent manner (Di Gennaro *et al*, 2003; Shintani *et al*, 2003a, 2003b) In addition, Iressa has been shown to have antimetastatic properties in human head and neck, and breast cancer cells (Mandal *et al*, 2002).

Four phase I studies (Baselga *et al*, 2002; Herbst *et al*, 2002; Ranson *et al*, 2002; Nakagawa *et al*, 2003) have evaluated gefitinib in more than 250 patients, of whom 28 had head and neck cancer. Gefitinib was well tolerated at doses from 150 to 800 mg m^−2^, the most frequent grade 1 or 2 toxicities being diarrhoea (47–55%), asthenia (44%) and an acneiform follicular rash (46–64%). Antitumour activity, including both partial responses (PRs) and cases of prolonged stable disease (SD), was observed at all doses. Clinically meaningful SD was achieved in 50% of patients with head and neck cancer, and quality of life (QoL) ratings also remained stable during treatment, except in one study where they improved significantly over time (LoRusso *et al*, 2003).

A phase II study has evaluated oral gefitinib (500 mg day^−1^) as first- or second-line monotherapy in 52 patients with recurrent or metastatic head and neck cancer most of whom had previously received combination chemotherapy or radiotherapy (Cohen *et al*, 2003a, 2003b). Of these, 47 patients were evaluable for tumour response and an objective PR rate of 10.6% (one complete response) was demonstrated. Disease control, defined as objective tumour response plus SD, was achieved in 53% of patients and was sustained for more than 6 months in 13% of patients. The response rates and survival times of patients who received gefitinib as first-line therapy were not significantly different to those of patients who had received prior chemotherapy. Overall, the median times to progression and death were 3.4 and 8.1 months, respectively, with an estimated 1-year survival of 29%. These results are more favourable than those achieved with chemotherapy in this setting, but with the additional benefit of reduced treatment-related toxicity. There was only a single case of grade 4 toxicity (hypercalcaemia), a 4–6% incidence of grade 3 toxicity (anorexia, diarrhoea, nausea and hypercalcaemia), grade 1 or 2 skin rash in 48% and grade 1 or 2 diarrhoea in 50%.

In July 2002, AstraZeneca plc opened an expanded access programme to provide single-agent gefitinib to patients with locally advanced, relapsed or metastatic head and neck cancer. Patients were eligible if they had failed standard treatment, or were not fit enough to receive other systemic anticancer therapy. We report our experience in using this agent in this patient group.

## PATIENTS AND METHODS

### Eligibility criteria

Patients with recurrent or metastatic head and neck cancer were recruited into this expanded access programme through the multidisciplinary head and neck clinics at the Royal Marsden and St George's Hospitals. Patients were eligible for recruitment if they had had a previous histologically or cytologically confirmed squamous cell cancer of the head and neck. Patients with recurrent and/or metastatic disease were eligible for treatment if they had received previous systemic chemotherapy and/or radiotherapy or were ineligible or unfit for such therapy. There was no requirement for patients to have a specific life expectancy. Patients were excluded if they were suitable for further radiotherapy, chemotherapy, or other systemic anticancer medication or had participated in a previous blinded study involving gefitinib. Patients with incomplete wound healing from prior oncological or other major surgery were also excluded. The presence of another significant clinical disorder or laboratory finding or evidence of clinically active interstitial lung disease was also considered as an exclusion criterion. In general, these inclusion criteria permitted entry of a number of patients with very poor prognosis disease.

### Treatment plan and dose modification

Gefitinib was initially administered by mouth at a total dose of 500 mg once daily. Patients who were unable to swallow were allowed to dissolve the tablets in water, and to have this solution delivered via nasogastric, gastrostomy or jejunostomy tube. Therapy was continued until disease progression, concomitant illness preventing further administration, unacceptable toxicity, or patient withdrawal. Dose reduction to 250 mg was permitted if toxicity became unacceptable to the patient with the option to increase the dose again if the toxicity improved. Toxicity was graded using the National Cancer Institute Common Toxicity Criteria version 2.0. These doses were selected in the absence of specific data on a dose–response relationship in head and neck cancer, although data from the randomised IDEAL1 study in patients with lung cancer demonstrated no difference between 250 and 500 mg (Fukuoka *et al*, 2003).

### Response assessment

Patients were assessed clinically and radiologically prior to commencing gefitinib and were subsequently assessed clinically on a monthly basis, with radiological assessment, where possible, at 8–12 weeks. Patients were imaged using the same modality as used at baseline. Radiological response guidelines as defined by the Response Evaluation Criteria in Solid Tumours (RECIST) were used, defining responses after at least 8 weeks of therapy as either a complete response (CR), a PR, SD or PD. Disease control was defined as the sum of patients achieving a CR, PR or SD. Confirmation of responses was required after 12 weeks. With respect to clinical response, the RECIST criteria were applied to visible or palpable areas of disease and CR, PR, SD or PD was recorded. In addition, an assessment of symptomatic response was made using a four-point scale previously reported in adenoid cystic carcinoma and non-small-cell lung cancer (Hardy *et al*, 1989; Hill *et al*, 1997). In this scale, individual symptoms were documented prior to treatment, and at subsequent assessment each symptom was recorded as being either (a) worse, (b) unchanged, (c) improved or (d) resolved. A subjective response in a particular symptom was defined as (c) or (d). The overall best symptom control was evaluated for each patient and if multiple symptoms responded differentially then the results were interpreted as follows: ‘no change’ + ‘worse’ scored ‘worse’; ‘no change’ + ‘better’ scored ‘better’; and ‘better’ + ‘worse’ scored ‘no change’. No attempt was made to measure QoL using a validated questionnaire for patients with head and neck cancer. All cases of clinical or radiological response or SD were reviewed by three observers.

### Statistical analysis

The primary end points were response rate and time to progression (TTP). Secondary end points included survival and toxicity. Time to progression and survival were measured from the date of commencing Iressa until disease progression or death, respectively, and were summarised by Kaplan–Meier curves. Data were updated to September 2004.

## RESULTS

### Prior therapy

A total of 47 patients were enrolled in the extended access programme from March 2003 to June 2004. Their characteristics and prior therapy are shown in Table 1. Gefitinib was administered orally in 46 cases (98%) and via a gastrostomy tube in one patient (2%). In all, 18 (38%) patients had received prior platinum-based chemotherapy in the context of radical chemoradiotherapy at some point in their treatment. Seven (15%) of these had also received further palliative systemic therapy (including platins, 5-fluorouracil, taxanes and triapine (a novel ribonucleotide reductase inhibitor)) prior to commencing gefitinib. Nine (19%) patients had received prior systemic therapy in a palliative context only, using the same agents, prior to commencing gefitinib. Only four (9%) patients received gefitinib as first-line therapy, the majority (35, 74%) receiving it as second- or third-line therapy, with eight (17%) receiving it as their fourth to seventh line of treatment. Follow-up continued after disease progression until death. Only three patients (6%) received subsequent systemic therapy, two with platinum-based chemotherapy and one within a phase I trial of systemic administration of an oncolytic virus.

### Response assessment

Treatment responses are summarised in Table 2. In terms of objective clinical response, four (8%) patients achieved a PR, and 13 (28%) had SD as their best response, such that the disease control rate was 36%. Response to treatment was not related to prior therapy. Those who responded all had locally recurrent disease at the time of commencing gefitinib (one in the nasopharynx, two in the region of the pinna and one in the floor of mouth). All four patients subsequently progressed locally with no evidence of systemic metastatic disease. Radiological assessment was performed in only 22 (47%) of patients, largely due to the rapidly progressive nature of the disease in the majority of the remaining patients. A total of 17 patients (36%) progressed and died prior to the 8-week assessment with a further three (6%) having been too unwell to attend their follow-up and imaging appointments. One patient had mucosal disease that was not assessable by imaging, and four patients did not undergo imaging despite having radiologically assessable disease. These results reflect the reality of treating a group of patients with end-stage head and neck cancer in whom performance status and disease activity can change rapidly. One PR was observed (2%), and 12 patients (26%) had SD, giving a radiological disease control rate of 28%. Of the patients with SD, seven had local recurrence at presentation, of whom five subsequently progressed locally with no evidence of metastatic disease and two have not yet progressed. Five patients had metastatic disease at the time of commencing gefitinib and all have since progressed. There was a reported improvement in symptoms in 16 (34%) patients and there was no change in a further five (11%) patients. There was symptomatic deterioration in 26 (55%) of patients.

### Survival data

**Figure 1:**
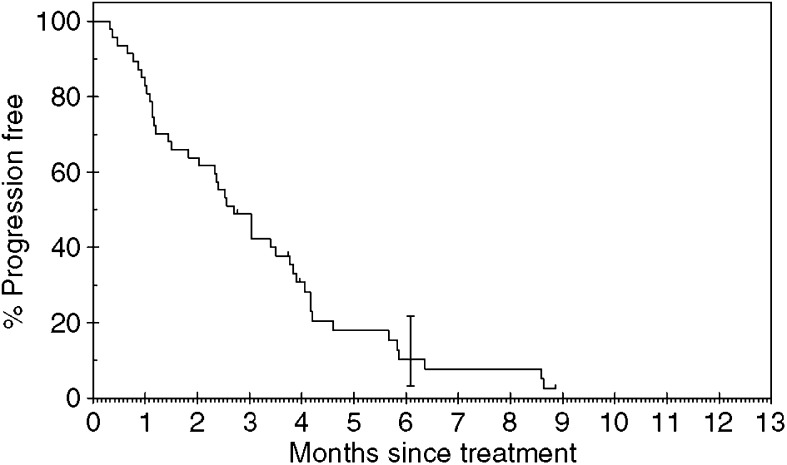
Progression-free survival.

**Figure 2:**
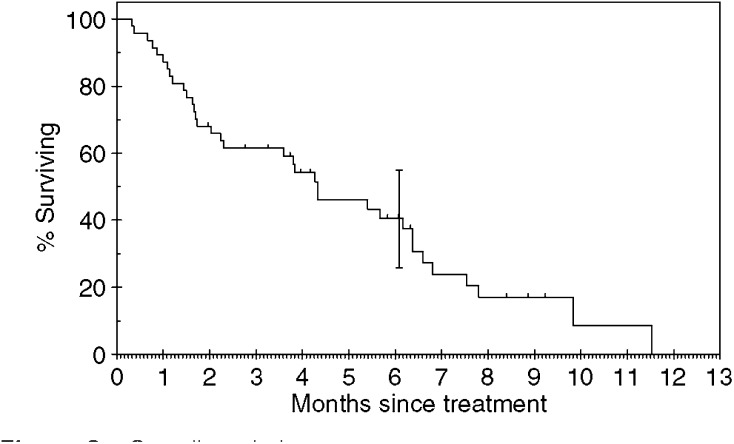
Overall survival.

In total, 44 of the 47 patients have developed PD. Three patients remain on gefitinib with SD. The median TTP was 2.6 months (range 0–9 months). The median survival was 4.3 months (range 0–13 months). The median follow-up time was 5 months (range 2–9 months). Of the 47 patients, eight were still alive at the time of analysis. Figures 1 and 2 show the Kaplan–Meier curves for progression-free and overall survival. Univariate analysis showed that stage of disease was the only significant factor affecting progression-free survival (see Table 3).

### Toxicity

Toxicity data and dose reductions are summarised in Table 4. In all, 34 patients were assessable for toxicity. Of the 13 (28%) patients in whom toxicities were not documented, nine died before their scheduled outpatient visit, one deteriorated and missed the scheduled visit, two were admitted to other hospitals and no toxicity was documented, and one patient was lost to follow-up.

Skin toxicity in the form of an acneiform folliculitis usually affecting the face (65% of those affected) and trunk (42% of those affected) was the most frequently observed side effect. Of the 26 patients affected, 20 experienced grade 1–2 skin reactions with only four patients experiencing a grade 3 reaction. Folliculitis was the commonest reason for dose reduction. Diarrhoea (grade 1–2) affected 16 (47%) of the patients in whom toxicities were recorded. Fatigue and anorexia were reported in a few cases, and one patient experienced plantar-palmar erythema. No patient experienced treatment-related lung toxicity and no grade 4 toxicity of any type was observed.

A dose reduction from 500 to 250 mg per day was made in 14 (30%) of the patients. In all but two of these patients (both with grade 2 folliculitis), the side effects improved. Gefitinib was stopped temporarily in three patients with folliculitis and after resolution of symptoms was restarted at a dose of 250 mg. One patient was admitted to another centre with an episode of supraventricular tachycardia of uncertain aetiology. In this patient, gefitinib was stopped and not restarted due to simultaneous demonstration of PD on imaging investigations.

## DISCUSSION

This is the second report on the use of gefitinib in locally advanced, recurrent or metastatic head and neck cancer and, despite the fact that it represents a single institutional study of relative small numbers of patients, we believe that it provides important further clinical information in this area. Owing to the nature of the extended access programme under which the drug was made available, there was no scope for inclusion of a control arm (placebo, best supportive care or alternative systemic therapy). This issue is currently being addressed in a randomised trial of gefitinib *vs* methotrexate.

The objective clinical response rate was only 8.4%, less than the radiologically confirmed 10.6% response rate reported in the only other published study of this agent in patients with head and neck cancer (Cohen *et al*, 2003a, 2003b) This study also reports a reduced median TTP (2.6 *vs* 3.4 months) and median survival (4.3 *vs* 8.1 months) compared to the previous report. The survival rate at 12 months was 29% in Cohen's series, compared to 0% in this study. The reasons for these differences may be explained in terms of the characteristics of the patients in the two studies. In general, the patients reported here represented a group with a very poor prognosis in whom palliative chemotherapy was not an option. The patients in our study were more likely to have locally recurrent disease (62 *vs* 44%) and have a poorer performance status (PS 0: 0 *vs* 21%, PS 1: 55 *vs* 62%, PS 2: 40 *vs* 17% and PS 3: 4 *vs* 0%). Many of our patients had rapidly progressing disease at the time of commencing gefitinib, as demonstrated by the number of patients who progressed before the first scheduled radiological assessment. More patients in Cohen's study had been fit to receive prior therapy, especially surgery (89 *vs* 51%) and chemotherapy (85 *vs* 63%), underscoring the fact that more of the patients recruited to our programme had never been fit enough to receive radical treatment.

The obvious attraction of using gefitinib in this group of patients (in the absence of other available therapeutic manoeuvres) was the ease of oral administration and the predicted lack of significant toxicity. In this study, the toxicity of palliative gefitinib was mild, although the folliculitis previously reported with gefitinib was more prevalent and florid than has been previously reported. Facial rash was a frequent cause of patients feeling self-conscious about their appearance and a rash affecting the trunk or limbs was frequently pruritic. The impact of the cutaneous rash on the QoL of patients has not been formally assessed in this study but it is noteworthy that the only reason for dose reduction in 11 patients was folliculitis, and that it was a contributory factor in another two patients. It has previously been suggested that the folliculitis associated with gefitinib may be a marker of treatment outcome (Perez-Soler, 2003). Our data provide some support for this conclusion in that four of the 14 patients who required a dose reduction had a PR and the other 10 had SD. However, in this group of patients, it is also possible that the association of response with skin rash is a reflection of the fact that patients who responded or who had SD were likely to have been taking the drug long enough to develop the side effect, whereas the nonresponders tended to stop taking gefitinib early. Diarrhoea and fatigue were mild and infrequent and generally managed by simple antidiarrhoeal medication. There was no evidence of pulmonary toxicity in this group of patients. Despite the apparent lack of activity of this agent, 21 (45%) patients reported stabilisation or improvement of disease-related symptoms on treatment. It is worth bearing this figure in mind when considering the use of gefitinib as a palliative therapy in patients with head and neck cancer. However, given that the objective response rate is inferior to that reported for platinum-based chemotherapy in this context, the latter should remain the standard of care in this setting for those patients who are fit enough to tolerate such therapy.

In the last 2 years a number of studies have begun to shed light on factors that predict the probability of response to gefitinib in patients with lung cancer. In particular, it appears that patients with mutations in exons 18–21 of the EGFR gene (corresponding to the ATP binding site) are more likely to respond to treatment (Paez *et al*, 2004). These mutations have been identified predominantly in patients with adenocarcinoma but similar mutations have been identified in two patients with squamous cell lung cancers (Kim *et al*, 2005). Recently, deletions in exon 19 have been reported in three of 41 Korean patients with squamous cell cancer of the head and neck (Lee *et al*, 2005). These discoveries have given renewed impetus to the prospect of using gefitinib (and related drugs) in patients with head and neck cancer. However, as with other targeted therapies, it is likely that if gefitinib does find a role it will be in combination with established therapies, such as chemotherapy and radiotherapy in patients with newly diagnosed disease. In addition to other agents that target the EGFR pathway (erlotinib, cetuximab), gefitinib will have to be considered alongside a plethora of novel agents directed against a variety of cellular targets. In particular, agents that target different growth factor receptors, the angiogenic switch and the apoptotic pathway appear to hold great promise in the treatment of head and neck cancer.

## Figures and Tables

**Table 1:** Patient characteristics

	**Patients**	
**Characteristic**	**No.**	**%**
Total	47	100
Male	35	74
Female	12	26
		
*Age (years)*		
Median	62	
Range	18–93	
		
*Disease at time of commencing IRESSA*		
Locally recurrent	29	62
Metastatic^a^	18	38
		
*Histology*		
Squamous cell carcinoma	45	96
UCNT^b^	1	2
Carcinosarcoma	1	2
		
*ECOG performance status*		
0	0	0
1	26	55
2	19	40
3	2	4
		
*Prior therapy (n*=*43*)		
Surgery	22	51
Radiotherapy	40	93
With chemotherapy	18	42
Alone (radical or palliative)	22	51
Chemotherapy	27	63
Radical chemoradiation only	11	26
Radical chemoradiation and then for recurrence or metastases	7	16
Only for recurrent disease or metastases	9	21

a These patients may also have had local recurrence simultaneously with metastatic disease.

b Undifferentiated carcinoma of nasopharyngeal type.

**Table 2:** Response

	***N***	**%**
*Clinical response (objective)*		
CR	0	0
Partial response	4	8
Stable disease	13	28
Progressive disease	30	64
		
*Radiological response (RECIST)*		
CR	0	0
PR	1	2
SD	12	26
PD	9	19
nd	25	53
		
*Symptom response (best)*		
(d) Resolved	0	0
(c) Improved	16	34
(b) Unchanged	5	11
(a) Worse	26	55

**Table 3:** Univariate analysis of (a) progession-free survival and (b) overall survival

	***n***	**Hazard ratio (95% CI)**	***P*-value**
*(a)*			
*Gender*			
Female	12	1	0.7
Male	35	1.15 (0.5–2.29)	—
			
*Age*			
Per year	47	1 (0.99–1.02)	0.75
			
*Line of therapy*			
First	21	1	0.35
Second+	26	0.75 (0.4–1.38)	—
			
*Stage*			
Local disease	11	1	0.03
Nodal±distant mets	36	2.52 (1.09–5.77)	
			
*PS*			
1	18	1	0.13
2 or above	29	1.6 (0.86–2.97)	—

*(b)*			
*Gender*			
Female	12	1	0.81
Male	35	1.1 (0.49–2.45)	—
			
*Age*			
Per year	47	1.01 (0.99–1.03)	0.41
			
*Line*			
First	21	1	0.06
Second+	26	0.51 (0.25–1.02)	—
			
*Stage*	11	1	0.27
Local disease	36	1.61 (0.69–3.73)	—
Nodal±distant mets			
			
*PS*			
1	18	1	0.1
2 or above	29	1.84 (0.89–3.82)	—

**Table 4:** Toxicity grades and dose reductions

			**Grade (no of patients affected)**					
**Toxicity**	**No.**	**%**	**0**	**1**	**2**	**3**	**4**	**n.s.**
No toxicities documented	13	30	—	—	—	—	—	—
								
*Of remaining 34 patients*								
Skin	26	76	8	9	11	4	0	2
								
Diarrhoea	16	47	18	12	4	0	0	—
								
Fatigue	5	15	29	2	3	0	0	—
								
Anorexia	2	6	32	2	0	0	0	—
								
Lung toxicity	0	0	0	0	0	0	0	—
								
*Dose reduction*								
Patients requiring dose reduction: *n*=14 (30%)								
Reasons:								
Folliculitis alone	11							
Folliculitis and diarrhoea	2							
Fatigue	1							
								
*Timing of dose reduction*:								
Eight patients at 2–6 weeks								
Five patients at 9–13 weeks								
One patient at 17 weeks								
Of 14 patients requiring dose reduction, four had had a partial response								
10 had had stable disease								
								
*Of patients receiving dose reduction, patients who had to discontinue therapy: n*=*4*								
Temporary cessation (2–3 weeks) due to folliculitis	3							
Permananent cessation following supraventricular tachycardia	1							

n.s., not significant.
